# *Trichoderma harzianum* Produces a New Thermally Stable Acid Phosphatase, with Potential for Biotechnological Application

**DOI:** 10.1371/journal.pone.0150455

**Published:** 2016-03-03

**Authors:** Amanda Araújo Souza, Vanessa Oliveira Leitão, Marcelo Henrique Ramada, Azadeh Mehdad, Raphaela de Castro Georg, Cirano José Ulhôa, Sonia Maria de Freitas

**Affiliations:** 1 Laboratory of Biophysics, Department of Cellular Biology, University of Brasília, 70910-900, Brasília, Brazil; 2 Laboratory of Enzymology, Department of Cellular Biology, University of Brasília, 70910-900, Brasília, Brazil; 3 Laboratory of Mass Espectrometry, Embrapa Recursos Genéticos e Biotecnologia – 70770-917, Brasília, Brazil; 4 Laboratory of Enzymology, Institute of Biology, University Federal of Goiás, 74001-970, Goiania, Brazil; CNRS, FRANCE

## Abstract

Acid phosphatases (ACPases) are produced by a variety of fungi and have gained attention due their biotechnological potential in industrial, diagnosis and bioremediation processes. These enzymes play a specific role in scavenging, mobilization and acquisition of phosphate, enhancing soil fertility and plant growth. In this study, a new ACPase from *Trichoderma harzianum*, named ACPase II, was purified and characterized as a glycoprotein belonging to the acid phosphatase family. ACPase II presents an optimum pH and temperature of 3.8 and 65°C, respectively, and is stable at 55°C for 120 min, retaining 60% of its activity. The enzyme did not require metal divalent ions, but was inhibited by inorganic phosphate and tungstate. Affinity for several phosphate substrates was observed, including phytate, which is the major component of phosphorus in plant foods. The inhibition of ACPase II by tungstate and phosphate at different pH values is consistent with the inability of the substrate to occupy its active site due to electrostatic contacts that promote conformational changes, as indicated by fluorescence spectroscopy. A higher affinity for tungstate rather than phosphate at pH 4.0was observed, in accordance with its highest inhibitory effect. Results indicate considerable biotechnological potential of the ACPase II in soil environments.

## Introduction

Phosphorus (P) is an essential nutrient for normal growth and metabolic processes in plants and microorganisms. It is also involved in the biosynthesis of cellular components, such as nucleic acids, phospholipids and proteins [1,2]. Over 80% of the phosphorus applied to soil is lost due to its broad adsorption, precipitation or conversion to organic form [3,4]. In alkaline soils, phosphorus is encountered fixed in the tricalcium phosphate salt and in acid soils as aluminum phosphate and iron phosphate [1,4]. The main mechanisms of P solubilization employed by soil microorganisms include: (1) solubilization and mineral dissolving of complex P compounds, (2) the release of P during substrate degradation and (3) liberation of extracellular enzymes, such as phosphatases for enzymatic degradation.

These enzymes are usually classified as alkaline phosphatases, acid phosphatases and protein phosphatases [3–5]. Acid phosphatase (ACPase) (orthophosphoric monoester phosphohydrolase EC 3.1.3.2) is a hydrolase that promotes monoester phosphate hydrolysis, transforming organic phosphate into a soluble inorganic form [5, 6]. Structural analysis of the ACPase from fungi has revealed a monomeric protein with two domains, one large with α-helix and β sheets (α/β strands) and another with a smaller α-helix domain [7]. The active site is located at the interface of the α/β domains, presenting fundamental arginine and histidine amino acid residues for enzymatic activity, with the conserved *RHGXRXP*motif [7, 8]. ACPases hydrolyze phosphomonoesters in a two-step process. First, the catalytic histidine makes a nucleophilic attack on the bound phosphomonoester, creating a phosphorylated intermediate; in the second step, a water molecule hydrolyses the phosphohistidine intermediate, releasing P [7, 8].

ACPases are widely distributed in organisms such as plants, animals and microorganisms, and have been characterized across diverse tissues such as seeds, roots, prostate and bone cells [9–14]. To date, they have been applied in a broad range of processes including scavenging, mobilization and acquisition of P, enhancement of soil fertility and plant growth [15]. In animal cells, they have been used as biomarkers in radioimmunoassays for diagnosis of bone metastases, chronic inflammation and prostate cancer [16, 17]. In industrial process, ACPases are applied in feed processing for monogastric animals releasing phosphate, for enhancement of nutritional value and in reduction of phosphate excreted by animals [18, 19]. Application in bioremediation of polluted soils has also been reported using ACPases derived from plants and mycorrhizal fungi [20]. Published data has also shown that ACPases from microorganisms are more efficient in hydrolysis of organic phosphate than those derived from plants [15].

A considerable number of ACPases have been characterized from fungi such as *Aspergillus sp*., *Humicola* sp., *Mucor* sp., *Penicillium* sp., *Metarhizium* sp. and *Trichoderma harzianum* [13, 21–25], with their potential in mediating availability of phosphorous to plants from organic compounds investigated by numerous groups [26, 27]. Of these fungi, *T*. *harzianum*is noteworthy, as a saprophytic fungus present in soils, including the Brazilian Cerrado biome. This fungus has been highlighted as a potent fertilizer agent, given its ability to release, induce uptake and provide soluble phosphate in the soil[15, 27]. Additionally, this fungus is able to colonize roots and leaves of host plants, promoting beneficial biochemical changes. The most important changes comprise increased abiotic stress, sequestration or solubilization of inorganic nutrients (carbon, nitrogen and phosphorus) and induction of resistance to diseases caused by plant pathogens [28, 29].

Given the considerable application of *T*. *harzianum* as a biological control agent, numerous studies have been conducted to increase our understanding of the mechanisms involved in mycoparasitism [30, 31]. It is well known that this fungus is capable of producing hydrolytic enzymes such as phosphatases, β-glucanase [32], chitinases [33], xylanases [34], and N-acetylglucosaminidase [35]. In addition, studies on phosphate deprivation have revealed the potential of *Trichoderma* spp. as phosphate solubilization agents, fixing phosphates present in the soil, and enhancing soil fertility and plant growth [15].

Leitão et al [25] purified and characterized an acid phosphatase (ACPaseI) from *T*. *harzianum*. This enzyme presented an optimum pH and temperature at 4.8 and 55°C, respectively, and was strongly inhibited by sodium tungstate. It was also characterized as a non-specific enzyme, catalyzing the hydrolysis of a wide range of phosphorylated esters, including organic molecules involved in cellular process, such as adenosine triphosphate (ATP), adenosine diphosphate (ADP) and adenosine monophosphate (AMP).

Considering the relevance and the potential biotechnological application of these phosphatases, efforts have been made to comprehend the molecular mechanisms and the relationship between structure and function. To date, understanding of uptake and phosphate release mechanisms in these enzymes remains unclear. In the present paper, we report the purification and functional characterization of a new enzyme presenting acid phosphatase activity from *T*. *harzianum*, named ACPase II.

## Materials and Methods

### Materials

Culture media reagents were purchased from Himedia (India). Reagents for protein assays were purchased from Bio-Rad^®^ (Quick Start Bradford, USA). Reagents for deglycosilation and substrates for enzymatic assays were purchased from Sigma Aldrich^™^ (USA).

### Microorganism and culture conditions

Conidiospores from a single spore-derived pure culture for strain *T*. *harzianum* ALL42 (HS574263.1, GenBank) were provided by the Department of Biochemistry and Molecular Biology, Enzymology Laboratory, Federal University of Goiás, Goiânia, Goiás, Brazil. A stock culture of the microorganism was recovered on Malt Yeast Glucose (MYG) medium containing 0.5% (w/v) malt extract, 0.25% (w/v) yeast extract, 1.0% (w/v) glucose and 2.0% (w/v) agar, with incubation at 28°C. A sexual conidia were isolated in sterile saline solution, following centrifugation at 3000 g.

For enzyme production, 10^7^ spores.ml^-1^of the fungus *T*. *harzianum* were inoculated in modified Minimal Medium (MM) containing 0.03% (w/v) CaCl_2_.6H_2_O, 0.14% (w/v) (NH_4_)_2_SO_4_, 0.03% (w/v) MgSO_4_.7H_2_O, 1.5% (w/v) glucose and 0.25% (w/v) yeast extract. Growth cultures were incubated in 500 ml Erlenmeyer flasks with constant shaking (180 rpm) at 28°C for 48 h. Fungal mycelium was harvested by filtration through filter paper (90 mm), filtrate dialyzed overnight against distilled water and freeze dried for use as a source of acid phosphatase.

### Enzymatic assay

Acid phosphatase activity was measured according to Leitão et al [25], using *p*-Nitrophenyl phosphate disodium salt hexahydrate (*p*-NPP) (Sigma Aldrich^™^) as substrate. One unit (1U) of acid phosphatase activity was defined as 1 μM of *p*-Nitrophenol (*p*-NP) formed per minute [36]. Protein concentration was determined by the Bradford method, using bovine serum albumin as standard [37].

### Purification of acid phosphatase and analysis of acid phosphatase purity

Crude extract containing enzymes was 10-fold concentrated by ultrafiltration using a 10 kDa membrane (Millipore, 63.5 mm). A 1 mg sample was applied onto a molecular exclusion Superdex 200 HiLoad 16/60 column (GE Healthcare), then equilibrated with 50 mM sodium acetate buffer, pH 4.8, using an AKTA Purifier fast protein liquid chromatography system (FPLC, GE Healthcare). Fractions presenting acid phosphatase activity were pooled and dialyzed against 50 mM sodium acetate buffer, pH 4.8,containing 3 M (NH_4_)_2_SO_4_ (buffer A). The samples were applied on a Hitrap Phenyl FF (High sub) column (GE Healthcare) previously equilibrated with buffer A. The column was washed with the same buffer and the bound proteins eluted using a decreasing gradient of 3 M to zero of (NH_4_)_2_SO_4_in the same sodium acetate buffer described above. Fractions containing acid phosphatase activity were pooled, dialyzed against water and stored at -20°C.

Polyacrylamide gel electrophoresis under denaturing conditions (12%SDS-PAGE) was carried out to estimate protein purity and the molecular mass of the purified enzyme [38]. Protein was silver stained as described by Blum [39] and molecular mass was estimated using molecular weight markers β-galactosidase (116 kDa), Bovine serum albumin (66.2 kDa), Ovalbumin (45 kDa), Lactate dehydrogenase (35 kDa) and REase Bsp98l (25 kDa) (Thermo Scientific). The error of the molecular mass determination was estimated based on analysis of data for five 12% SDS-PAGE runs, with linear regression calculated using Image Lab 5 (Bio-Rad).

### Protein identification byMALDI-TOF/TOF spectrometry

The band corresponding to the enzyme identified as ACPase II was excised from polyacrylamide gels and digested with trypsin Gold-Mass V582A (Promega), according to Schevchenko et al [39]. The resulting peptides of each spot were submitted to mass spectrometric analyses using an UltraFlexIII MALDI-TOF/TOF (Matrix-Assisted Laser Desorption/Ionization-Time of Flight), controlled with Flex Control 3.0 software (Bruker Daltonik). The sample was mixed with α-cyano-4hydroxycinnamic acid matrix solution (3:1, v/v) directly applied onto an MTP AnchorChip 400/384 target plate (Bruker Daltonik) and dried at room temperature. Peptides presenting mono isotopic masses were obtained in reflector mode with external calibration using the Protein Calibration Standard (Bruker Daltonik). Peptide MS/MS spectra were obtained by means of LIFT fragmentation. The software Flex Analysis 3.0 (Bruker Daltonik) and PepSeq (Waters) were used for mass spectrometric data analysis. Peptide primary structures were inferred by means of manual interpretation of fragmentation. The obtained sequences were then searched against the NCBI_nr_ protein database (www.ncbi.nlm.nih.gov) using the algorithm Blastp. The identified protein sequences were analyzed using the Peptide Mass tool from the ProtParam Server (www.expasy.org) in order to predict theoretical molecular weight [40].

### Deglycosylation of the acid phosphatase

The purified enzyme was treated with the enzymatic protein Deglycosylation kit, according to the manufacturer’s protocol (Sigma Aldrich^™^). An ACPase sample containing 100 μg of protein was added to the reaction buffer and denaturation solution and boiled for 5 minutes. The sample was then incubated for 3 hours at 37°C with PNGase F and O-glycosidase. The deglycosylated protein was analyzed by12% SDS-PAGE, as described above.

### Biochemical characterization of the purified ACPase II

The effect of pH on ACPase II activity was determined by varying the pH from3.2 to 5.6 using 50 mM glycine-HCl (pH 3.2–3.4) and 50 mM sodium acetate (pH 3.6–5.6) buffer. The effect of temperature on the enzymatic activity was determined at pH 3.8over a temperature range from 35 to 80°C. Thermostability was determined by pre-incubation of the purified enzyme at pH 4.0, 50, 55 and 60°C for 120 min and returning the temperature to60°C (optimum temperature). Kinetics parameters were estimated using *p*-NPP at concentrations ranging from 0.033 to 1.0μM. Activities were determined by the standard procedure and kinetics parameters (K_m_ and V_max_) calculated from the fitted Michaelis-Menten curve by nonlinear regression/enzymatic kinetics/Michaelis-Menten enzyme kinetics via the program GraphPad Prism (version 6.01, 2000). The ability of ACPase II to hydrolyze different substrates (Sigma Aldrich^™^), i.e. *p*-NPP, ATP, ADP, AMP, fructose 6-phosphate, glucose 1-phosphate, phenyl phosphate, β-glucose phosphate and phytic acid, was evaluated at substrate concentrations of 5 mM [41]. The effects of inorganic phosphate and sodium tungstate on ACPase II activity were also determined after pre-incubation of purified enzyme with the respective compound (5 mM) for 10 min at 60°C.

### Fluorescence Spectroscopy Assays

Fluorescence measurements of 0.66 μM ACPase II were performed at pH 4.0–5.5 (10 mM sodium acetate buffer), pH 6.5–9.0 (10 mM Tris buffer), and 25°C using the Jasco FP-6500 Spectrofluorimeter (Jasco Analytical Instruments, Tokio, Japan) coupled to a Jasco ETC-273T Peltier system (Jasco Analytical Instruments) with water circulation. Both the excitation and emission slits were fitted at 10.0 nm, with excitation and emission wavelengths at 295 nm and 300–400 nm, respectively.

ACPase II spectral changes were investigated in the presence of sodium phosphate (0–660 μM) and sodium tungstate (0–660 μM) with fluorescence quenching as a function of pH 4.0, 7.0 and 8.5. The averages of four fluorescence spectra were recorded and processed using the program Spectra Manager (Jasco Analytical Instruments, Tokio, Japan). The fluorescence emissions were fitted according to the classic Stern-Volmer equation (Eq 1) as follow [42]:

Fo/F=1 + Ksv[Q] (1)

where F and F_0_ representthe fluorescence intensities in the presence and absence of quencher, respectively, *K*_*SV*_ is Stern-Volmer constant and [Q] is the concentration of the quencher.

In order to calculate the dissociation constants for ACPase-related phosphate and tungstate complex, the equilibrium between free and bound molecules is assumed to be proportional to the fluorescence intensity as [B]/[B_o_] ∝ F/F_o_ [43]. The dissociation constants (K_D_) for the equilibrium between free and bound ions on ACPase II were calculated by nonlinear regression, again using GraphPad Prism (version 6.01, 2000).

### Statistical analysis

All experiments were performed in triplicate. Statistical analysis in enzymatic assays was performed based on standard deviation. Differences were considered to be significant at a value of p <0.05.

## Results and Discussion

### Acid phosphatase production by *T*. *harzianum*

*T*. *harzianum*, *Neurospora crassa*, *Apergillus niger*, and *Humicola lutea* are recognized as efficient producers of hydrolytic enzymes, such as β-glucanase [32], chitinases [33], xylanases [34], N-acetylglucosaminidase [35] and acid phosphatases [8, 22, 25,44]. Among such enzymes, ACPases have gained special attention due to their broad biotechnological potential across in industry, diagnostics, bioremediation, mobilization and acquisition of P, enhancement of soil fertility and plant growth [15, 45–48]. Secretion of ACPases in producing species of fungi is stimulated by acidic pH and specific carbon sources, with inhibition in the presence of inorganic phosphate [22, 43, 49, 50]. In previous studies, we showed that *T*. *harzianum* produced ACPases in medium supplemented with several carbon sources, such as glucose, cell wall of *Rhizoctonia solani*, *Macrophomina phaseolina*, *Fusarium* sp. and *Sclerotinia sclerotorum* [25, 51, 52].

**Fig 1:**
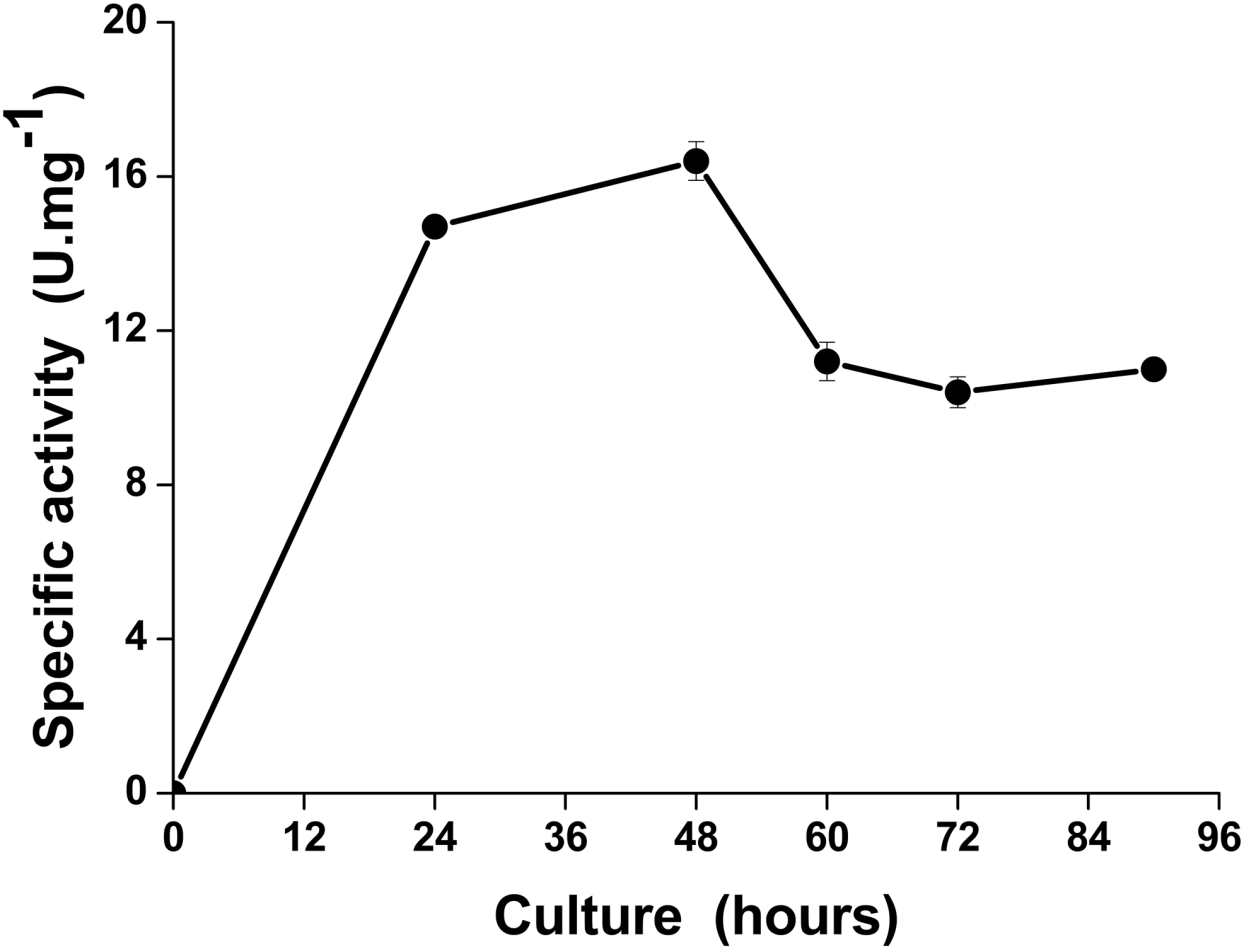
Time-course of extracellular acid phosphatase production by *Trichoderma harzianum*. The strain was grown on 1.5% glucose at 28°C and pH 4.0 without inorganic phosphate. The experiment was conducted in triplicate and differences were considered to be significant at a value of p <0.05.

In this work, the amount of 10^7^ spores.ml^-1^ of *T*. *harzianum* was cultivated in medium supplemented with glucose (15 g.L^-1^) without the addition of inorganic phosphate (pH adjusted to 4.0) for 90 hours. The highest levels of ACPases (14.3 U.mg^-1^) were obtained from culture supernatants at 48 hours (Fig 1). Additionally, a decrease of ACPases secretion in the presence of inorganic phosphate was observed.

### Purification and characterizationof ACPase II from *T*. *harzianum*

**Fig 2:**
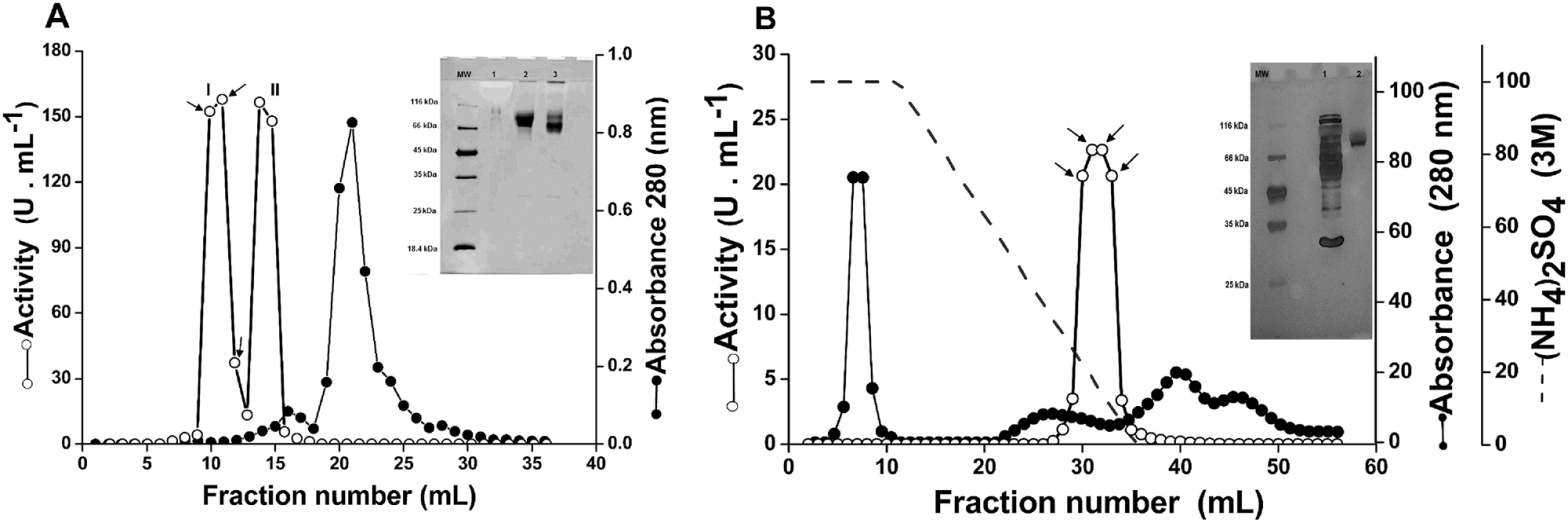
Purification and activity profiles of ACPase II from *T*. *harzianum*. **(A)** Molecular exclusion chromatography profile of *T*. *harzianum* crude extract on a Superdex S-200 column eluted with 50 mM acetate buffer, pH 4.8, 150 mM NaCl (inset 12% SDS PAGE of peak I; MW: molecular weight; lane 1: fraction 9, lane 2: fraction 10, and lane 3: fraction 11, indicated by the arrows). **(B)** Hydrophobic chromatography of fraction 9–11 using a Hitrap Phenyl FF column. The eluted bound proteins from fractions 31–34 show a phosphatase activity (single peak) corresponding to the ACPase II. The elution of the bound proteins was carried out with a decreasing gradient of 50 mM acetate buffer, pH 4.8 containing 3 M (NH_4_)_2_SO_4_. Inset, 12% SDS PAGE of pooled fractions 31–34 containing ACPase II; MW- molecular weight; lane 2: crude extract; lane 3: ACPase II from fraction 31–34, presenting the higher activity, as indicated by the arrows. The experiment was conducted in triplicate, with differences considered to be significant at a value of p <0.05.

In most reports on fungal ACPase, two or more chromatography steps are generally required for protein purification [13–14, 21, 23, 24, 53]. Similarly, a new acid phosphatase from *T*. *harzianum*, named ACPase II, was purified in our study by using two steps. Firstly, two distinguishable acid phosphatase activity peaks (I and II) (Fig 2A) from crude extract of enzymes, corresponding to the fractions 9–11 and 14–15, were obtained by size exclusion chromatography using a Superdex 200 column on an AKTA Purifier (GE Healthcare).

The activity found in peak I (Fig 2A and inset) corresponded to the eluted ACPase II (fractions 9 to 11) and that from peak II corresponded to the eluted ACPase I (fractions 14 to 15), as purified by Leitão et al [25]. This was assumed based on the differences in molecular mass of ACPase I (58 kDa) and ACPase II (90 kDa), as previously estimated through 12% SDS-PAGE (S1A Fig), together with respective activities presented in a zymogram gel (S1B Fig). Secondly, the ACPase II was purified by hydrophobic chromatography (HC) in Hitrap Phenyl FF, as shown in Fig 2B. Both resins preserved the biological activity of ACPase II, resulting in a 21.7-fold purification, with overall yield of 43.3% and specific activity of 1030.0 U.mg^-1^. A starting material of 200 ml of *T*. *harzianum* protein crude extract (0.175 mg/ml) was concentrated to 3.314 mg in 1ml and applied onto a superdex 200 column (Table 1).

**Table 1:** Summary of the purification steps for ACPase II from *T*. *harzianum*.

Step	Total protein (mg)	Total activity (U)	Specific activity (U.mg^-1^)	Purification (fold)	Yield (%)
Crude enzyme	3.314	157	47.4	1.0	100
Superdex 200	0.405	134	330.8	7.0	85.3
Hitrap Phenyl FF	0.066	68	1,030	21.7	43.3

The molecular mass of ACPase II was estimated by 12% SDS-PAGE (Fig 2B) due its self-association tendency in solution, as indicated by size exclusion chromatography and dynamic light scattering (data not shown). ACPase II was eluted in a void volume and presented hydrodynamic radius of 14.3 nm and molecular mass of 326 kDa, compatible with a tetramer.

SDS-PAGE analysis revealed a purified ACPase II of 90 ± 5 kDa (Fig 2B, inset), distinct to previously characterized acid phosphatases from fungi such as *Mucor hiemalis* (45 kDa), *Metarhizium anisopliae* (44 kDa), *Aspergillus fumigatus* (18 kDa), *Neurospora crassa* [23, 24, 54, 55] and *Aspergillus caespitosus* (186 and 190 kDa) [13].

**Fig 3:**
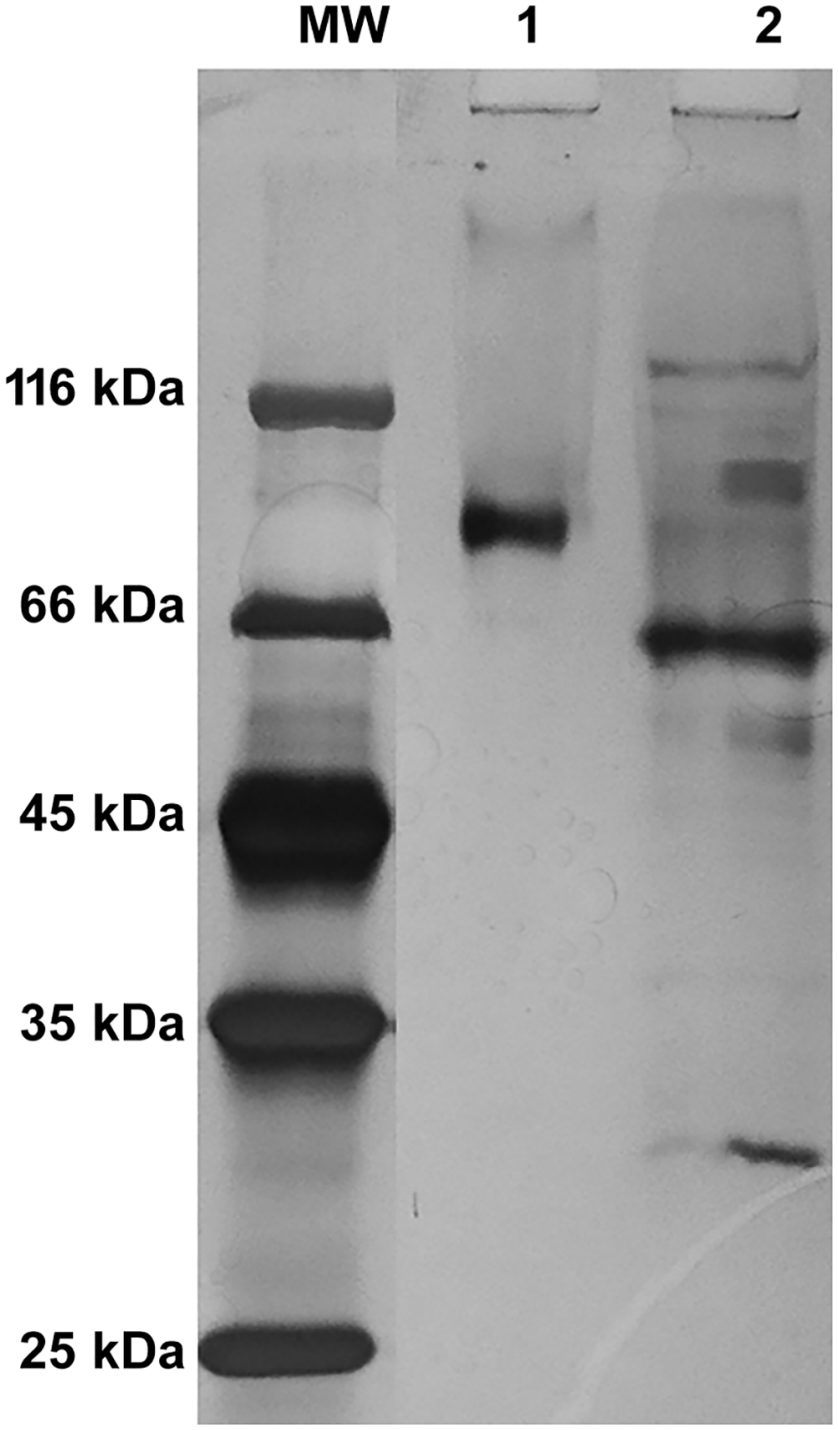
Glycosylation profile of the purified ACPase II from *T*. *harzianum*. After ACPase II (100 μg) deglycosylation the electrophoresis was carried out using a 12% cross-linked polyacrylamide gel. MW: molecular weight (Thermo Scientific); Lane 1: ACPase II intact (86 μg); lane2: ACPase II after deglycosylation; MW: Molecular mass marker proteins. ACPase II appears as a higher molecular mass of 90 ± 5.0 kDa.

In order to assess whether ACPase II is a glycoprotein, the purified enzyme was treated with PNGase and O-glucosidase. After SDS PAGE, a defined band was obtained corresponding to a protein of 58 ± 5 kDa, according to the almost of predicted values presented in the S1 Table. The enzyme was characterized as a glycoprotein, composed of approximately 30% carbohydrate (Fig 3). Analysis of the peptide sequence using NetNGlyc 1.0 and NetOGlyc 3.1 showed two potential N-glycosylation and four potential O-glycosylation sites (S2B Fig).

### ACPase II sequencing by Maldi-Tof/Tof spectrometry

Identification and classification of the enzyme was carried out based on the amino acid sequence of peptides obtained from protein hydrolysis with trypsin, using MALDI-TOF/TOF spectrometry (S2A Fig). Seven peptides were sequenced and compared to sequences from GenBank. The amino acid sequences and molecular mass matched phytases from *T*. *harzianum*, histidine acid phytase from *Trichoderma pleuroticola* and a hypothetical protein from *Trichoderma virens* available on the NCBI database (S1 Table and S2B Fig). It is important to note that all of histidine phytase are refereed as histidine acid phosphatase and, to date, no sequence of acid phosphatase from *T*. *harzianum* has been found in the NCBI database. The molecular mass observed through SDS-PAGE (Fig 2B) did not match any theoretical molecular weight from similar sequences, indicating post-translational modifications, such as the identified N-glycosylation and O-glycosylation sites (Fig 3 and S2B Fig), or partial sequences of this protein on the database. However, after deglycosilation with PNGase F and O-glycosidase, the ACPase II shows a molecular mass of 68 ± 5 kDa, according to the predicted value (S1 Table). The sequence of peptides derived from ACPase II displayed a conserved histidine composing the catalytic site, with approximately 98% and 96% homology with 3-phytase from *T*. *harzianum* and histidine phytase from *T*. *pleuroticola*, respectively (S2B Fig).

### Biochemical characterization of ACPase II

**Fig 4:**
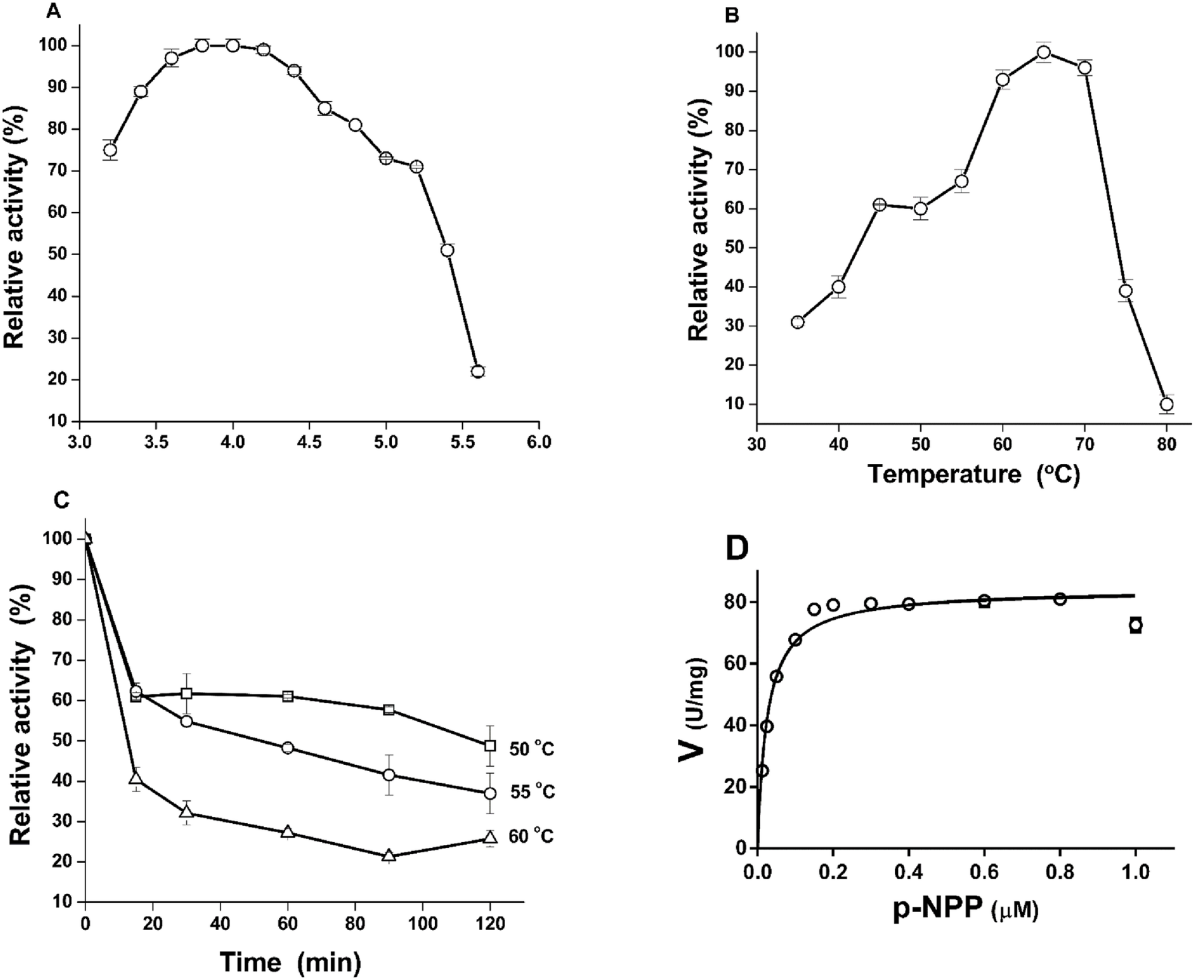
Biochemical properties of the purified ACPase II from *T*. *harzianum*. **(A)** Optimum pH was estimated using 50 mM glycine HCl and sodium acetate buffer ranging from 3.2 to 5.6. **(B)** Optimal temperature of 60°C was estimated from 35 to 80°Cand 50 mM sodium acetate buffer pH 3.8 was used. **(C)** Thermal stability was analyzed by previously incubating the enzyme at 50, 55 and 60°C in 50 mM sodium acetate buffer pH 3.8 for 120 min. **(D)** Enzymatic kinetics fitted according to Michaelis-Menten approximation using *p*-NPP at concentrations ranging from 0.033 to 1.0 μM. K_m_ and V_max_ were 0.027 μM and 83 U/mg, respectively. The enzymatic assays were conducted in triplicate and differences were considered to be significant at a value of p <0.05.

The optimum pH of ACPase II was 3.8 (Fig 4A), in agreement with data observed for fungal acid phosphatases from *M*. *anisopliae* [24], *A*. *niger* [56], *A*. *oryzae* [57] and *A*. *nidulans* [58], but distinct to those with optimum pH of 2.5 from *A*. *ficuum* [59] and *A*. *fumigatus* [55]. The optimum temperature of 65°C (Fig 4B) was similar to that for ACPases from *M*. *hiemalis* [23] and *A*. *niger* [55], but lower than observed for ACPases from *M*. *anisopliae* [24] and *A*. *caespitosus* [13]. Thermostability of ACPase II was analyzed as a criterion for potential of the characterized enzymes from this fungus in industrial application. ACPase II was stable for at least 120 min at 50°C and 55°C respectively, retaining 60% of maximum activity at 50°C and 45% of maximum activity at 55°C (Fig 4C).

ACPase II showed a greater affinity for *p*-NPP compared to ACPase I [25] and other ACPases from *Penicillium chrysogenum* (111 μM), *M*. *hiemalis* (434 μM), *A*. *bisporus* (370 μM) and *A*. *caespitosus* (29 μM) [13, 21,23, 60], based on K_m_ and V_max_ values of 0.027 μM and 83.93 U/mg, respectively (Fig 4D and Table 2). As ACPase II was not significantly inhibited by metal chelators, such as EDTA, the enzyme does not appear to require metal divalent ions as cofactors for catalytic activity (Table 3). In addition, the enzyme was not inhibited by sodium tartrate, was partially inhibited by inorganic phosphate and strongly inhibited by sodium tungstate (Table 3). Inorganic phosphate is a common inhibitor of most ACPases from fungi and in some cases is associated with mechanisms involved in enzyme regulation [13, 21–23, 55, 60]. Tungstate is a specific inhibitor of tyrosine phosphatase [24]. In both cases, the inhibition of ACPase II seems to be related with conformational changes of the protein resulting in steric hindrance surrounding of the active site. Fluorescence quenching provides evidences for this protein conformational change, as discussed below.

**Table 2:** Summary of the biochemical properties of the ACPase II from *T*. *harzianum*.

Biochemical properties	ACPase II
pH optimum	3.8 ± 0.01
Temp optimum (°C) 15 min	60.0 ± 0.02
Thermal stability/Relative activity (%)	
pH 3.8/50°C/ 60 min	61.0% ± 1.0%
pH 3.8/55°C/ 60 min	48.0% ± 1.0%
pH 3.8/60°C/ 60 min	27.0% ± 1.0%
k_m_	0.027 μM
V_max_	83.93 U/mg
Inhibition by inorganic phosphate	65.0% ± 2.0%
Inhibition by sodium tungstate	98.0 ± 1.0%

**Table 3:** Effect of different substrates and compounds on ACPase II activity.

Substrate	Relative activity (%)
*p*-NPP	100.0 ± 1.0
Phenyl sodium phosphate	52.3 ± 2.0
D-Glucose-1-phosphate	49.4 ± 1.0
ADP	39.0 ± 1.0
β-Glucose sodium phosphate	35.5 ± 1.0
D-Fructose-6-phosphate	33.0 ± 2.0
AMP	30.0 ± 2.0
ATP	25.3 ± 2.0
Phytic acid	20.5 ± 1.5
**Compound /Inhibitor**	
EDTA	137.6 ± 1.0
Sodium tartrate	114.4 ± 2.0
Mg^2+^	100.5 ± 2.0
Ca^2+^	93.5 ± 2.0
Inorganic phosphate	33.0 ± 2.0
Sodium tungstate	1.0 ± 1.0

Affinity of ACPase II for different substrates was observed, which may reflect a broad range of physiological functions in fungi, mainly associated with survival and environment adaptation. Additionally, ACPase II was not phosphate-specific, since it was able to cleave a broad range of phosphate esters, including *p*-NPP, a general substrate for acid phosphatases, ATP, ADP and AMP (Table 3). ACPase II was more efficient in cleaving phenyl sodium phosphate, D-glucose-1-phosphate, ATP, ADP, and AMP.

In summary, in contrast to ACPase I, based on the biochemical features, ACPase II from *T*. *harzianum* is an enzyme belonging to the acid phosphatase families, with higher activity than known ACPases from different sources. Potential practical applications of this purified phosphatase from *T*. *harzianum* may include bioremediation, biocontrol, plant nutrients uptake, aggregate stability, and carbon cycling and sequestration, as also observed with other fungal phosphatases [30, 31, 51, 52]. However, for these biotechnological applications, among those biochemical features, the structural characterization is important to be investigated.

### Structural characterization of ACPase II

**Fig 5:**
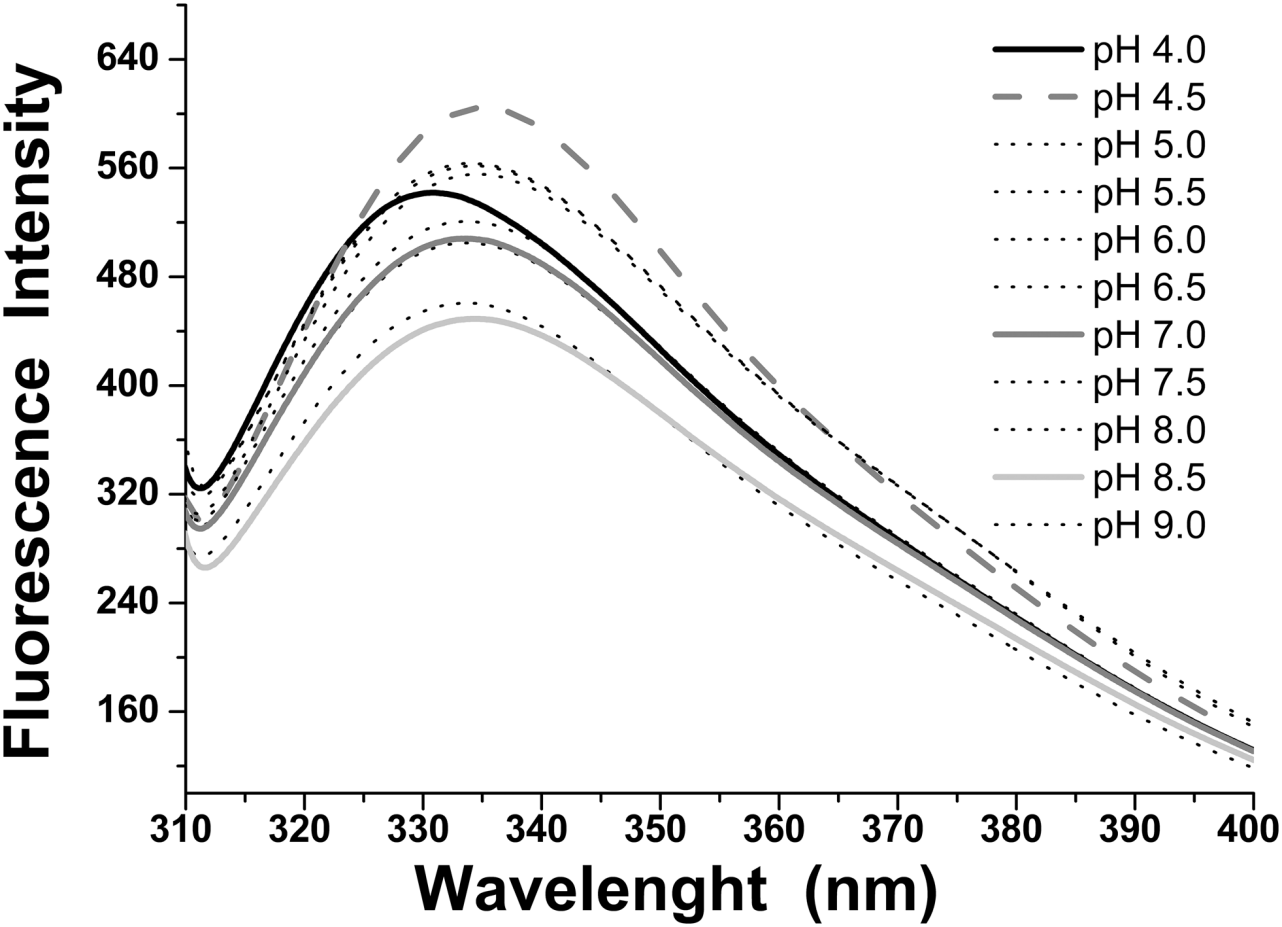
Fluorescence spectra of ACPase II as a function of different pHs (4.0–9.0). The fluorescence spectra were shifted by approximately 10 nm to shorter wavelength (336 to 327 nm) from pHs 4.5–9.0 to pH 4.0.

The structural proprieties of ACPase II were assessed by fluorescence in order to investigate the protein structure/function relationship. The fluorescence spectra of ACPase II as a function of pH were to a shorter wavelength (336 to 327 nm), from pHs 4.5–9.0 to pH 4.0. This data is compatible with changes on tryptophan residues from exposed to buried environments as a function of acidic pH. The intensity of emission bands were also very pH dependent, increasing or decreasing in conformity with changes in the solvent polarity surrounding the tryptophan residues. This is likely due to the ionization of charged side chains and their molecular reorientation, leading to groups of spectra between pH 5.0–5.5, 6.0–7.0, 7.0–9.0 (Fig 5). These results indicate that ACPase II is more stable at pH 4.0, when compared with others pHs. It is interesting to note that this structural feature is related to the differences in enzyme activities, with highest values observed at pHs ranging from 3.8 to 4.0 (Fig 4A).

### Effects of tungstate and phosphate ions on ACPase II structure and activity

The strategy employed in this work relied initially on purification and biochemical characterization of ACPase II from *T*. *harzianum* and its structural properties at pH 4.0. Indeed, we investigated the inhibitory effect of reactive ions, as tungstate and phosphate, correlated to structural properties. Inhibition of ACPase II by tungstate and phosphate observed at different pHs, was likely due to an inability of the substrate to occupy its active site. Tungstate and phosphate are polyatomic ions containing, respectively, an oxoanion of tungsten and phosphorus surrounded by four oxygen atoms which form several non covalent interactions in proteins, especially electrostatic interactions. Due their chemical properties, these compounds inhibit ACPase II (Table 3) from making electrostatic contacts, thus leading to inaccessibility of substrate for active site.

A fluorescence quenching assay was used to investigate conformational changes of ACPase II as a function of pH and affinity of compounds to ACPase II. This technique is a well-established tool that allows detection of conformational changes in proteins and protein-ligand interactions by monitoring solvent accessibility and the microenvironment of tryptophan residues under different conditions [61, 62]. Specifically, changes of tryptophan emission spectra occur in response to protein conformational changes, protein-protein association, ligand binding, denaturation, etc, in which affect the environment surrounding the side chain of this residue [42].

**Fig 6:**
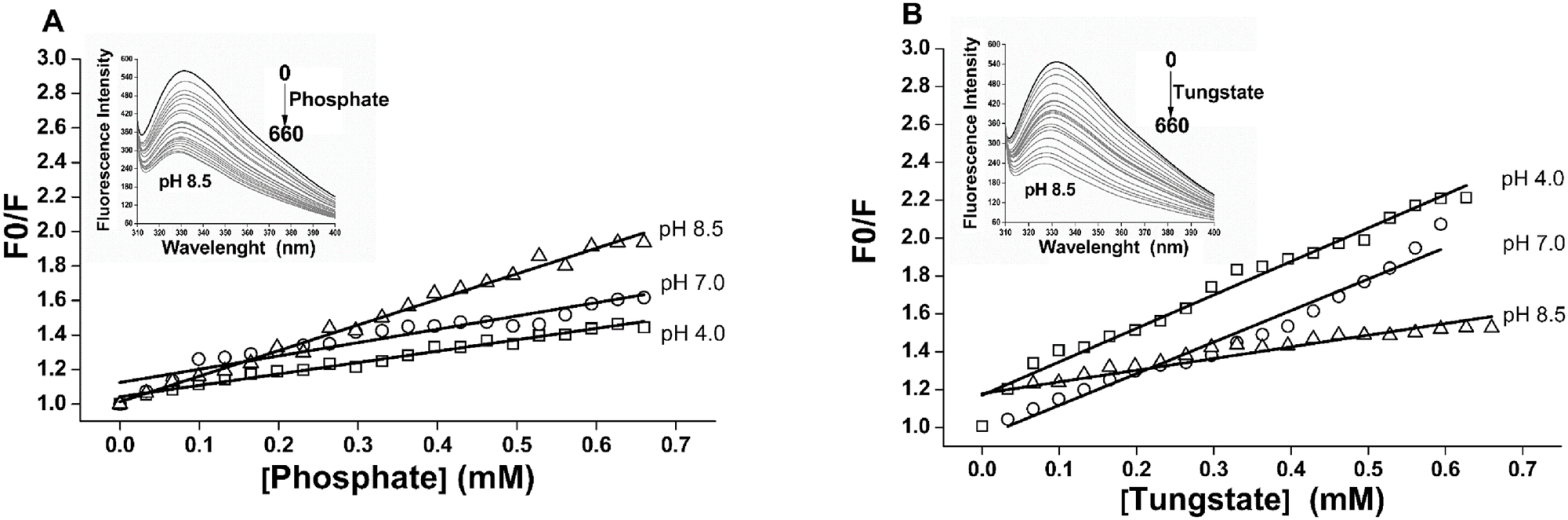
Stern—Volmer plots of ACPase II at different pHs in the presence phosphate and tungstate at 25°C. **(A)** The emission spectra as a function of increasing concentrations of phosphate (inset) led to a progressive reduction in the fluorescence intensity of tryptophan residues. **(B)** The emission spectra as a function of increasing concentrations of tungstate (inset) led to a progressive reduction in the fluorescence intensity of tryptophan residues. The *K*_*sv*_ values were presented in Table 4.

**Fig 7:**
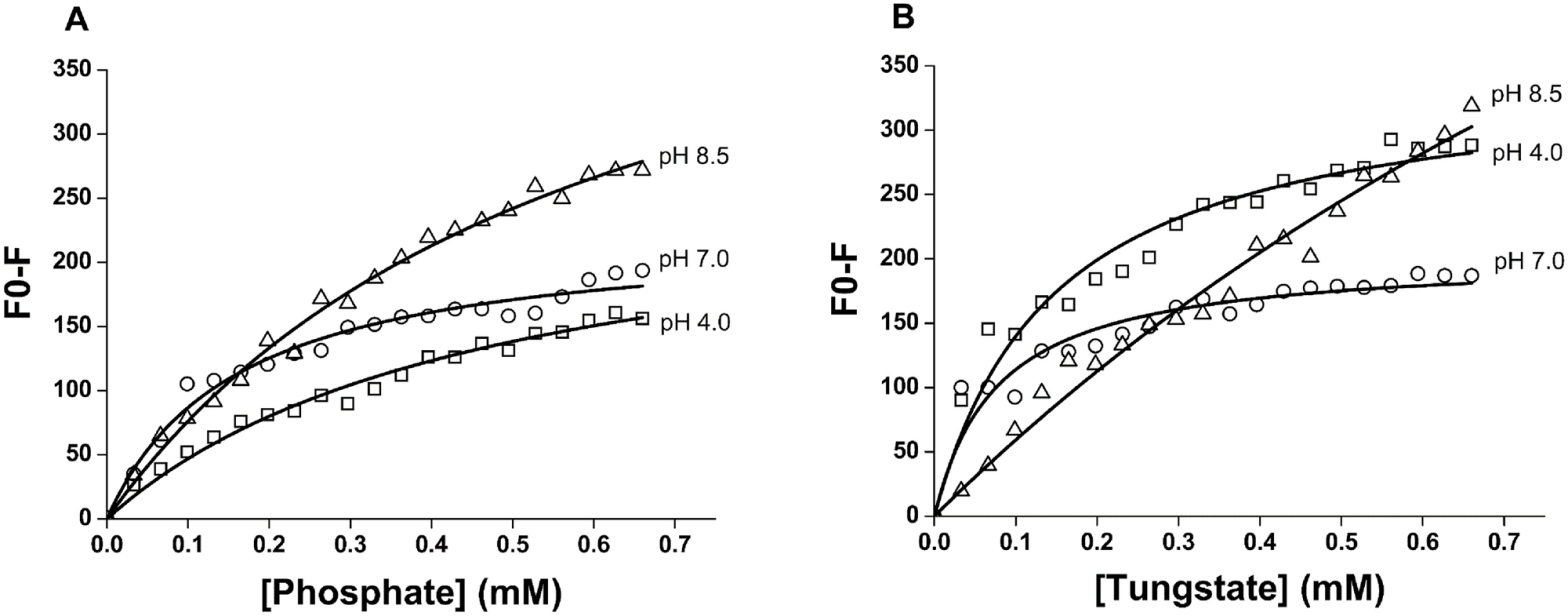
The spectral properties of the ACPase II in the presence of phosphate and tungstate monitored by fluorescence quenching at 330 nm, in different pHs. **(A)** The increasing concentrations of phosphate ranging from 0 to 660 μM led to a progressive reduction in the fluorescence intensities.**(B)** The increasing concentrations of tungstate ranging from 0 to 660 μM led to a progressive reduction in the fluorescence intensities. These data were considered in estimation of the dissociation constant (*K*_*D*_), as presented in Table 4.

Here in, the addition of increasing concentrations of tungstate and phosphate to ACPase II resulted in progressive decrease of fluorescence intensity at 330 nm, pH 4.0, 7.0 and 8.5 (data shown for pH 8.5; Fig 6A and 6B, inset), that were considered to estimate Stern-Volmer and dissociation constants (Figs 6 and 7). The Stern-Volmer plot profile and Ksv constants were consistent with a static fluorescence quenching process, as consequence of pH and protein-oxoanion complex formation. Phosphate and tungstate inhibitors binding to ACPase II also induce spectral shifts to shorter wavelength (from 332 nm to 328 nm) as seen at pH 8.5 (Fig 6A and 6B, inset), indicating the tryptophan changing from exposed to buried environment.

According to the Stern-Volmer adjustment, a linear correlation was observed for compounds at different pHs (Fig 6A and 6B), supporting the existence of a static quenching process involving ACPase II, phosphate and tungstate complex formation. Fluorescence quenching corresponded to a single population of tryptophan residues in the ACPase II [42]. These results may be due to electrostatic, as well as unknown interactions in the vicinity of the fluorophore in the presence of these compounds that were responsible for conformational changes of the protein [63].

Tungstate showed a greater access to ACPase II at pH 4.0 and 7.0 than at pH 8.5, as indicated by *K*_*sv*_ values (Table 4). In contrast, phosphate showed greater *K*_*sv*_ values at pH 8.5 than in acidic and neutral conditions. It is noteworthy that the residual charge of the protein as a function of pH resulted in conformational changes that do not conceal the tryptophan residues at pH 7.0 and 8.5, with an exception at pH 4.0 (Fig 5). These conformational changes at pH 7.0, as well as tryptophan microenvironment changes at pH 4.0, allowed for easy access of tungstate, although not at pH 8.5. On the other hand, they were responsible for the easy access of phosphate at pH 8.5. Comparatively, increase in pH leads to a greater protein conformational change that interferes in the access of tungstate and facilitates the access of phosphate.

**Table 4:** Stern—Volmer and dissociation constants of ACPase II by forming the enzyme-phosphate and tungstate complexes, at different pHs.

	Phosphate				Tungstate			
pH	Ksv (x10^2^) M^-1^	R^2^	K_D_ (x10^-4^) M^-1^	R^2^	Ksv (x10^2^)M^-1^	R^2^	K_D_ (x10^-4^) M^-1^	R^2^
4.0	6.60 ± 0.20	0.98	4.76 ± 0.61	0.97	17.66 ± 0.64	0.97	1.47 ± 0.20	0.93
7.0	7.70 ± 0.56	0.98	1.60 ± 0.18	0.96	16.64 ± 0.76	0.96	1.05 ± 0.11	0.94
8.5	14.81 ±0.34	0.98	5.96 ± 0.60	0.98	6.15 ± 0.51	0.94	18.20 ± 5.6	0.97

In order to calculate the dissociation constant of the ACPase II in complex with phosphate and tungstate, the equilibrium between free (Bo) and bounded (B) protein was assumed to be proportional to the fluorescence intensity, as B/Bo ∝ F/Fo [42, 43]. Fo is the initial fluorescence intensity and F is the fluorescence intensity in the presence of ligand (Table 4 and Fig 7).

The exact mechanism by which tungstate and phosphate inhibit ACPase II is still not understood. However, the binding of these compounds to ACPase II likely promotes protein low conformational changes, as indicated partially by the fluorescence quenching assay and a blue shift of emission bands. These findings are supported by buried rather than exposed tryptophan residues in ACPase II (blue shift of the emission band at ~328 nm) that were only accessed by occurrence of electrostatic barriers for tungstate at pH 8.5 and for phosphate at pH 4.0 and 7.0. However, *K*_*D*_ values are only slightly different for phosphate, suggesting that the microenvironment around the buried tryptophan presents low perturbations upon ion binding (Table 4).

As also observed, the values for *K*_*sv*_ for tungstate at pH 8.5 are very different. It is an indicative of differences in enzyme structure mainly concerning the ionized charged amino acid residues, such as histidine, which affect the tryptophan environment. All these results are associated with the highest affinity of the enzyme for tungstate at pH 4.0 and 7.0 and for phosphate at pH 8.5 (Table 4).

Considering the overall results of this study, tryptophan residues may be involved in the active sites of the enzyme, participating both in the binding and/or hydrolysis of the substrate and in the maintenance of the integrity of the active site. Additionally, fluorescence quenching shows that affinity of tungstate is higher than phosphate at optimum pH 4.0, in accordance with its inhibitory effect on ACPase II.

## Conclusion

*T*. *harzianum* is a filamentous fungus with considerable biotechnological potential in industrial processes and applications in sustainable agriculture. The data reported here reveal such biotechnological application, with ACPase II from *T*. *harzianum* appropriate for the removal of phosphates from several substrates including phytate, an important molecule present in soil environments and in many plant species that are used for animal feed. Potential also exists for the development of strategies for ACPase II that enables maximal enzymatic rates, efficient production through heterologous expression, and increased thermal stability.

## Supporting Information

S1 Fig 12% SDS-PAGE analysis and enzymatic activities in non-denaturing electrophoresis of the acid phosphatase I and acid phosphatase II from *T*. *harzianum*. **(A)** Lane 1: Crude extract from *T*. *harzianum* (80 μg); Lane 2: The purified ACPase II (27μg); Lane 3: ACPase I (85μg); MW-molecular weight (Thermo Scientific). Molecular masses were estimated using an Image Lab 5 (Bio-Rad) with values at 90 ±5 and 58 ± 2 kDa, respectively. **(B)** 8% PAGE of the enzymes incubated with 50 mM sodium acetate buffer (pH 3.8) and stained with substrate 4- methylumbelliferyl phosphate at 40°C. Lane 1 and 2: Crude extract from *T*. *harzianum* (10 μg and 45μg, respectively) presenting ACPase I and ACPase II activities, indicated by the arrows.
(PDF)

S2 Fig Sequencing and identification of the ACPase II. **(A)** MALDI-TOF/TOF mass spectrometry profile ms/ms of the purified peptides generated after hydrolysis of ACPase II with trypsin. **(B)** Sequence alignment of the seven peptides from ACPase II compared to those sequences from GenBank. The amino acids indicated by a bold box are the theoretical sites of glycosylation of the ACPase II predict by NetNGlyc 1.0 and NetOGlyc (www.expasy.org). A partial motif forming the catalytic active site is indicated by the arrow.
(PDF)

S1 Table Peptide sequences from hydrolyzed ACPase II obtained by MALDI-TOF/TOF and Blastp searches against the NCBI_nr_ protein database. (DOCX)
