# Supplementary figures and images for: Trichoderma harzianum Produces a New Thermally Stable Acid Phosphatase, with Potential for Biotechnological Application
Source: PLoS One. 2016 Mar 3;11(3):e0150455. doi: 10.1371/journal.pone.0150455 (PMC4777480; doi:10.1371/journal.pone.0150455)

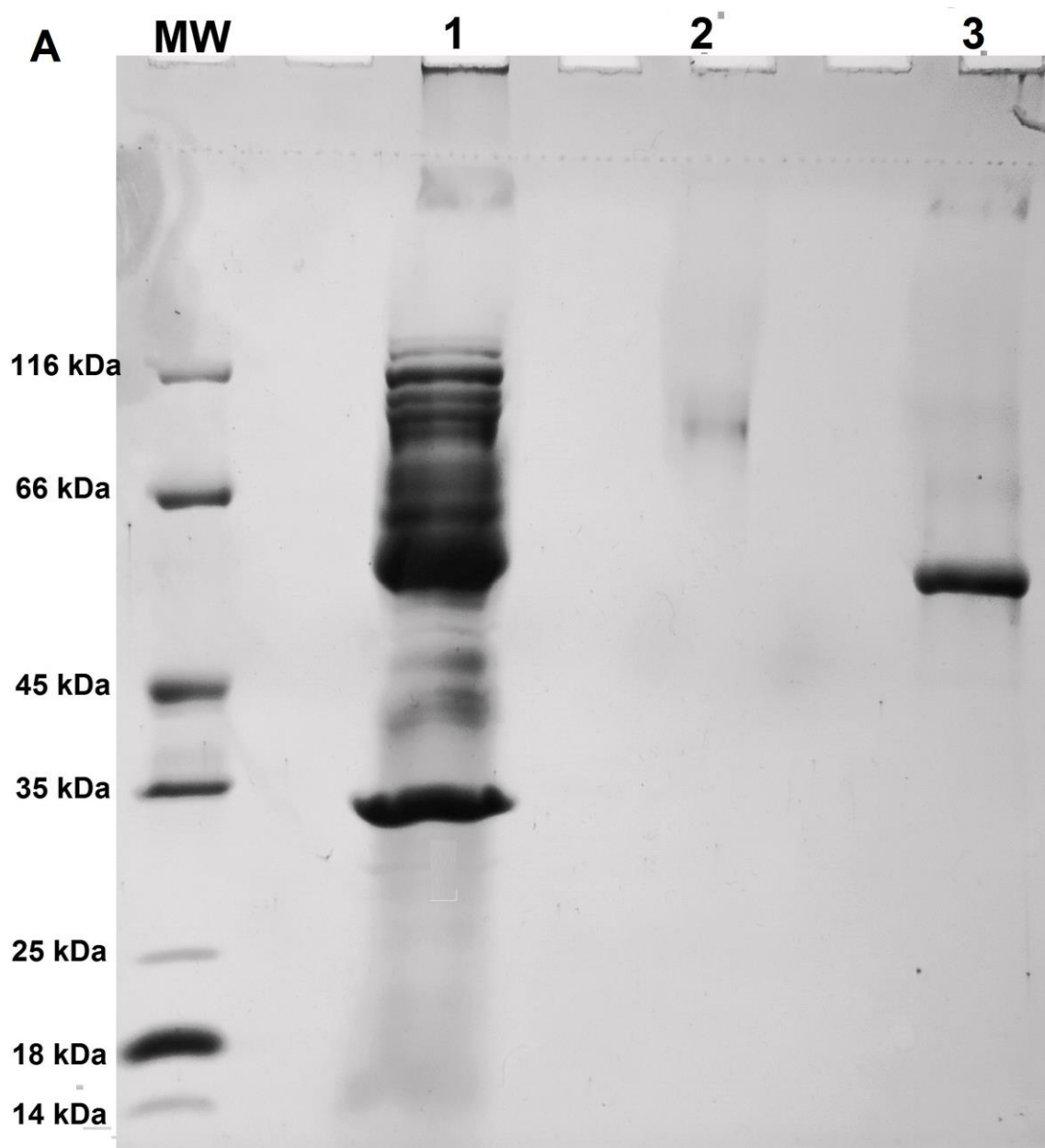

**B**

**1**

**2**

ACPase II  
→

ACPase I  
→

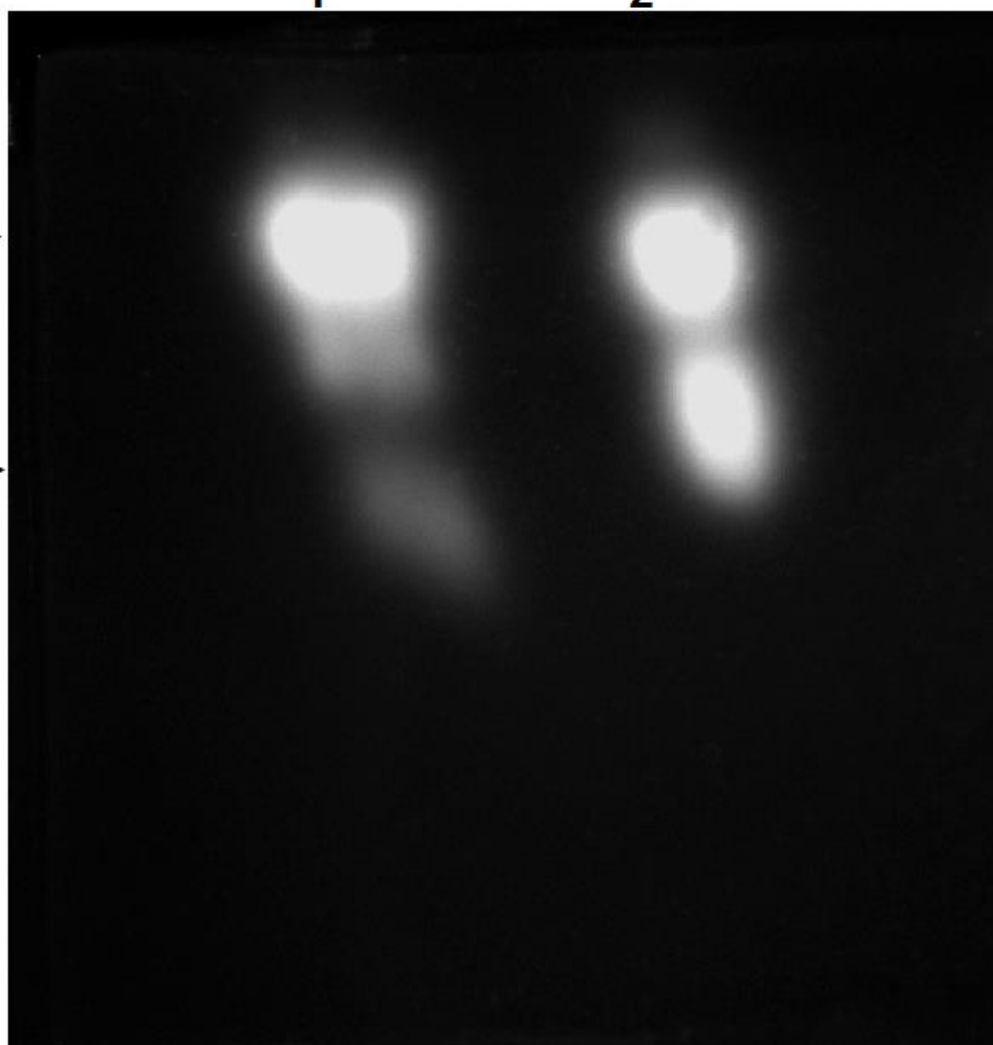

Supplement: S1 Fig — (A) Lane 1: Crude extract from T. harzianum (80 μg); Lane 2: The purified ACPase II (27μg); Lane 3: ACPase I (85μg); MW-molecular weight (Thermo Scientific). Molecular masses were estimated using an Image Lab 5 (Bio-Rad) with values at 90 ±5 and 58 ± 2 kDa, respectively. (B) 8% PAGE of the enzymes incubated with 50 mM sodium acetate buffer (pH 3.8) and stained with substrate 4- methylumbelliferyl phosphate at 40°C. Lane 1 and 2: Crude extract from T. harzianum (10 μg and 45μg, respectively) presenting ACPase I and ACPase II activities, indicated by the arrows. (PDF) [file pone.0150455.s001.pdf]
