# Supplementary material for: Trichoderma harzianum Produces a New Thermally Stable Acid Phosphatase, with Potential for Biotechnological Application
Source: PLoS One. 2016 Mar 3;11(3):e0150455. doi: 10.1371/journal.pone.0150455 (PMC4777480; doi:10.1371/journal.pone.0150455)
Supplement: S2 Fig — (A) MALDI-TOF/TOF mass spectrometry profile ms/ms of the purified peptides generated after hydrolysis of ACPase II with trypsin. (B) Sequence alignment of the seven peptides from ACPase II compared to those sequences from GenBank. The amino acids indicated by a bold box are the theoretical sites of glycosylation of the ACPase II predict by NetNGlyc 1.0 and NetOGlyc (www.expasy.org). A partial motif forming the catalytic active site is indicated by the arrow. (PDF) [file pone.0150455.s002.pdf]

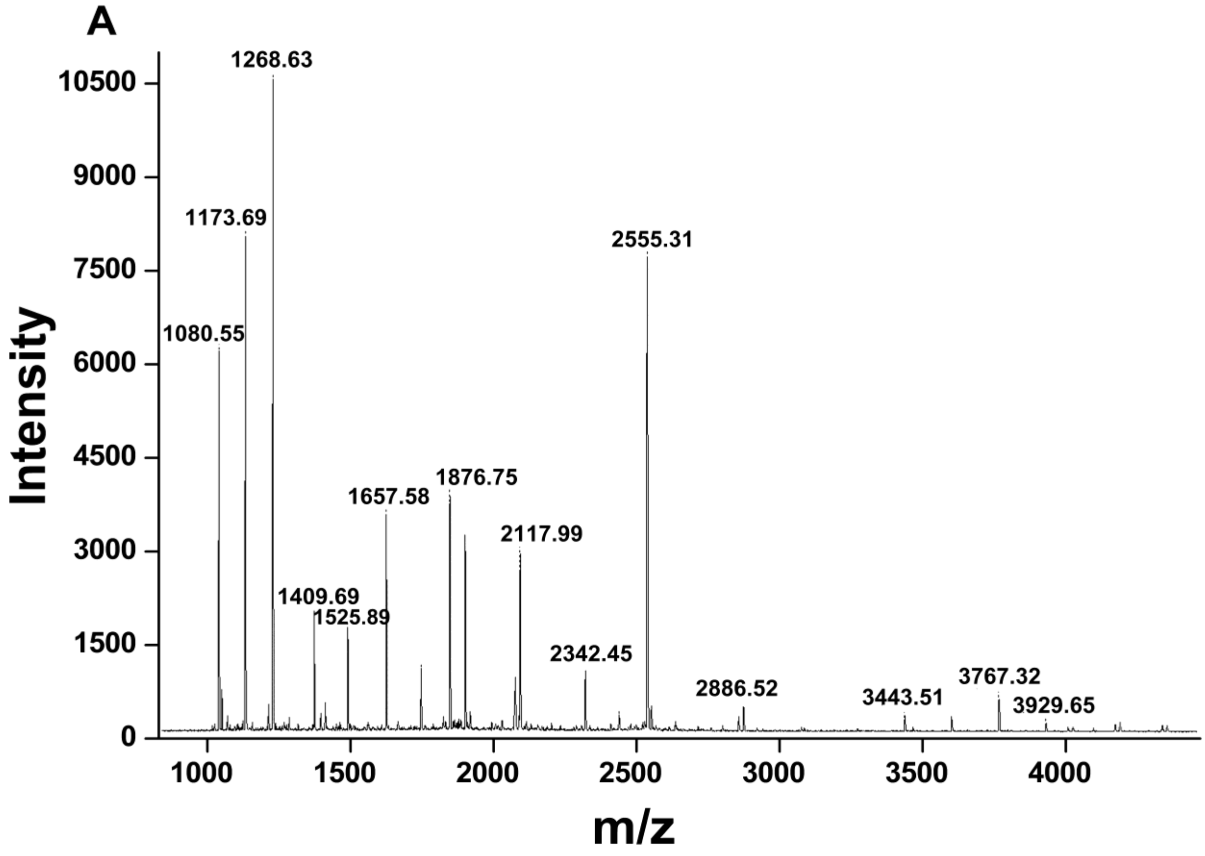
A
Intensity
m/z
1268.63
1173.69
1080.55
2555.31
1657.58
1876.75
2117.99
1409.69
1525.89
2342.45
2886.52
3443.51
3767.32
3929.65
0
1500
3000
4500
6000
7500
9000
10500
1000
1500
2000
2500
3000
3500
4000

B

```
T. pleuroticula 25483164        GYLEEFVAR
T. virens 358386411             GYLQEFVAR
T. atroviride 358395039         GYLQEFVAR
T. harzianum 84619439           GYLQEFVAR
T. harzianum 219816575          GYLQEFVAR
T. harzianum ALL42 Pep 1        GYLQEFVAR
                                 ***:***
```

```
T. pleuroticula 25483164        IGSTVATEFAL
T. virens 358386411             IGSTVATKFAA
T. atroviride 358395039         LGAELLTPFGR
T. harzianum 219816575          IGSTVATEFAL
T. harzianum 84619439           IGSTVATEFAL
T. harzianum ALL42 Pep 2        LGAELLTPFGR
                                :*: : * *.
```

```
T. pleuroticula 25483164        LTAFNLTALFQ-
T. virens 358386411             LTAFNLTRLFQ-
T. harzianum 219816575          LTAFNLTALFQ-
T. harzianum 84619439           LTAFNLTALFQ-
T. atroviride 358395039         SQNFNLGVAYRQ
T. harzianum ALL42 Pep 3        SQNFNLGVAYRQ
                                   ***   ::
```

```
T. pleuroticula 25483164        YNNGPGSPVAAAQG
T. virens 358386411             YNNGPGSPVAAAQG
T. harzianum 219816575          YNNGPGSPVAAAQG
T. harzianum 84619439           YNNGPGSPVAAAQG
T. atroviride 358395039         YPTSGAAPATFAQK
T. harzianum ALL42 Pep 4        YPTSGAAPATFAQK
                                * .. .:*.: **
```

```
T. pleuroticula 25483164        KADGLCSFDHVVSILQK
T. virens 358386411             SADGLCSFDHVVSVLQK
T. atroviride 358395039         NSDGLCSFDHVVSVLQK
T. harzianum 84619439           NADGLCSFDHVVSVLQK
T. harzianum 219816575          NDDGLCSFDHVVSVLQK
T. harzianum ALL42 Pep 5        NADGLCSFDHVVSSLQK
                                . ********** ***
```

```
T. atroviride 358395039         FLVNDAVVPIAESYHGCPE
T. pleuroticula 25483164        FLVNDAVVPISDSYHGCPK
T. virens 358386411             FLVNDAVVPISDSYHGCPK
T. harzianum 84619439           FLVNDAVVPISDSYHGCPK
T. harzianum 219816575          FLVNDAVVPISDSYHGCPK
T. harzianum ALL42 Pep 6        FLVNDAVVPISDSYHGCPK
                                **********::******:
```

```
T. pleuroticula 25483164        IVPFATHFTTQILECPAHKPTR
T. virens 358386411             IVPFATHFTTQILECPALKPTR
T. atroviride 358395039         IVPFATHFTTQILECPAYKPTR
T. harzianum 84619439           IVPFATHFTTQILECPAQKPTR
T. harzianum 219816575          LVPFATHFTTQILECPAQKPTR
T. harzianum ALL42 Pep 7        IVPFATHFTTQILECPAQKPTR
                                :**************** ****
```
