# Supplementary material for: Trichoderma harzianum Produces a New Thermally Stable Acid Phosphatase, with Potential for Biotechnological Application
Source: PLoS One. 2016 Mar 3;11(3):e0150455. doi: 10.1371/journal.pone.0150455 (PMC4777480; doi:10.1371/journal.pone.0150455)
Supplement: S1 Table — (DOCX) [file pone.0150455.s003.docx]

**S1 Table. Peptide sequences from hydrolyzed ACPase II obtained by MALDI-TOF/TOF and Blastp searches against the NCBI_nr_ protein database**.

| **Peptide sequence** | **Protein name** | **e-value** | **Molecular Mass (kDa)** |
| --- | --- | --- | --- |
| GYLQEFVAR | Phytase (*T. harzianum*) ACL37341.1 | 1.3 | 34.61 |
| LGAELLTPFGR | Histidine phosphatase domain (*T. virens)* EHK24007.1 | 0.069 | 58.39 |
| SQNFNLGVAYR | Histidine acid phytase (*T. pleuroticola*) ACT83166.1 | 0.019 | 56.83 |
| YPTSGAAPATFAQK | Histidine acid phytase (*T. pleuroticola*) ACT83166.1 | 1.00E-04 | 56.83 |
| NADGLCSFDHVVSSLQK | Phytase (*T. harzianum*) ACL37341.1 | 4.00E-05 | 34.61 |
| FLVNDAVVPISDSYHGCPK | Histidine acid phytase (*T. pleuroticola*) ACT83166.1 | 7.00E-11 | 56.83 |
| IVPFATHFTTQILECPAQKPTR | Histidine acid phytase (*T. pleuroticola*) ACT83166.1 | 3.00E-13 | 56.83 |

Molecular mass was estimated using the ProtParam server (www.expasy.org).
